# Case of Amputation of the Thigh for a Compound Fracture of the Candyles of the Femur

**Published:** 1838-11-21

**Authors:** Henry H. Smith

**Affiliations:** Resident Surgeon


*Case of Amputation of the Thigh for a compound fracture of the candyles of the femur.*

Samuel C——, æt. thirty-two years, was admitted, October 2d, 1838, for a compound fracture of the thigh. Whilst riding on some rail road cars in the neighbourhood of the city, he slipt, and was jammed between the one he was on and the one following, or fell under the car,—could not tell which. He was brought to the hospital the same evening. On his admission there was found a lacerated wound, an inch and a half long, on the inside of the thigh, about two inches above the patella, merely through the integuments, separating them from the fascia underneath, and extending from the saphena vein round to near the back part of the thigh. Considerable venous hæmorrhage occurred, probably from the saphena, as the wound was directly in its course. A small piece of bone, a half inch square, was found adherent to the skin, just above the upper edge of the wound, and there was also a small lacerated wound of the inside of the leg, and an excoriation on the outside of the other foot. The wound was drawn together by adhesive strips, lint wet with the white of egg applied over these, with the view of forming an artificial scab, and the limb bandaged and placed in a fracture box. Considerable hæmorrhage took place in the night, which was stopt by a compress over the saphena vein below the knee. Ordered laud. gtt. xxx.

*October 3d.*—Dosed through night; has not much pain; great heat and swelling around the knee-joint; little fever; pulse soft; skin pleasant; diet, beef tea, and the following mist.: R. G. assafœt. ʒij., tinct. opii. ʒiss., aquæ ℥iv., ft. mist. ℥ss. q. h. h. In the afternoon a consultation of the surgeons of the house was held, and agreed to meet to re-examine the limb next morning; dressings and diet continued.

*4th.*—Slept well; no pain; apparent disposition in wound to unite; symptoms good; water drawn off by cathether, owing to his being unable to pass it whilst on his back. Consultation ordered antiphlogistic treatment to the part.

*9th.*—Since last date has been doing well; no great heat about the joint; little pain; slight fever; sleeps well; wound sloughing at the edges; tongue moist, but slighly furred. Continue treatment.

*22d.*—From the last date wound has done remarkably well; the granulations look healthy, and the sloughs of the integuments are separating; there is considerable inflammation around the joint, with some fever.

*29th.*—Sloughing has continued since last date, more or less; patient has night sweats, and hectic fever; knee-joint opened by the sloughs, and the parts above the wound are much inflamed; dressed with poultice and bran in a fracture box, to catch the discharge; tenit-sapo camphorat applied to parts above the wound. Full diet and porter.

From this time, until November 5th, there was no material change, when an abscess formed and opened on the outer side of the knee; diarrhœa came on, and the patient's constitution was becoming seriously affected by hectic. A consultation of the surgeons, Drs. Harris, Randolph, and Norris, decided, therefore, on amputation; which was performed by Dr. Harris on November 7th, by the common circular method.

*November 14th.*—The stump was dressed to-day for the third time; the upper edge has united, and the whole surface is healthy. The patient's constitution has improved since the operation, and he is now doing well.

A dissection of the limb, after the operation, showed a fracture between the two candyles of the femur, extending up to an oblique fracture of its shaft, and the piece of bone spoken of as found at the upper part of the wound, was a piece of the internal candyles, which had been forced up from the pieces on each side of it.

